# Non-invasive respiratory volume monitoring identifies opioid-induced respiratory depression in an orthopedic surgery patient with diagnosed obstructive sleep apnea: a case report

**DOI:** 10.1186/s13256-015-0577-9

**Published:** 2015-04-29

**Authors:** Eamon Fleming, Christopher Voscopoulos, Edward George

**Affiliations:** 1 Respiratory Motion, 411 Waverley Oaks Rd #150, Waltham, MA 02452 USA; 2 Brigham and Women’s Hospital, 75 Francis St, Boston, MA 02115 USA; 3 59 Hawk Crest Court, Roseville, CA 95678 USA; 4 Massachusetts General Hospital, Harvard Medical School, 55 Fruit Street, Boston, MA 02114 USA

**Keywords:** Minute ventilation, Non-invasive, Obstructive sleep apnea, Opioid-induced respiratory depression, Post-operative, Respiratory volume monitoring

## Abstract

**Introduction:**

Obstructive sleep apnea and opioid-induced respiratory depression can unpredictably threaten respiratory competence in the post-anesthesia care unit. Current respiratory monitoring relies heavily on respiratory rate and oxygen saturation, as well as subjective clinical assessment. These assessments have distinct limitations, and none provide a real-time, objective, quantitative direct measurement of respiratory status. A novel, non-invasive respiratory volume monitor uses bioimpedance to provide accurate, quantitative measurements of minute ventilation, tidal volume and respiratory rate continuously in real time, providing a direct measurement of ventilation.

**Case presentation:**

The case describes an orthopedic surgery patient (54-year-old Caucasian man, body mass index 33.7kg/m^2^) with diagnosed obstructive sleep apnea in whom the respiratory volume monitor data depicted persistent apneic behavior undetected by other monitoring. The monitor was able to detect a sudden reduction in minute ventilation after initial opioid administration in the post-anesthesia care unit. The patient had sustained low minute ventilation until discharge. Neither respiratory rate data from the hospital monitor nor oxygen saturation readings reflected the respiratory decompensation, remaining within normal limits even during sustained low minute ventilation.

**Conclusions:**

The events of this case illustrate the limitations of current respiratory rate monitoring and pulse oximetry in the evaluation of post-surgical respiratory status. Our patient displayed stable respiratory rate and no evidence of desaturation, despite sustained low minute ventilation, and he received opioids in the post-anesthesia care unit despite already compromised ventilation. Because the available monitoring did not indicate the patient’s true respiratory status, he was treated with additional opioids, markedly increasing his risk for further respiratory decline.

## Introduction

Management of obstructive sleep apnea (OSA) is a growing concern in peri-operative care. It is estimated that one in four men and one in ten women have OSA, with the prevalence among surgery candidates exceeding that of the general population [1]. Population-based studies suggest that patients presenting for orthopedic surgery with OSA are at greater risk for post-operative pulmonary complications [2,3]. OSA is also substantially under diagnosed [4,5], and increasing evidence suggests that classic indicators such as age, body mass index and patient sex may in fact have little correlation with OSA prevalence or the manifestation of apnea post-operatively [6,7]. As a result, assessing individual patient risk for apnea can be difficult.

Disordered breathing is not the only threat to respiratory competence in the post-anesthesia care unit (PACU). In addition to the effects of surgical insult and anesthesia, narcotics administered for the management of pain can lead to opioid-induced respiratory depression (OIRD) or, as described by the Anesthesia Patient Safety Foundation, opioid-induced ventilatory insufficiency (OIVI) [8,9]. The potential synergy between unpredictable apneic and hypopneic events and OIVI is a significant threat to patient safety. Care providers typically rely on a combination of oxygen saturation, respiratory rate (RR) and subjective clinical assessment to evaluate respiratory status in the PACU, but these are only surrogate indicators of ventilatory drive. Without a direct, objective measurement of ventilation, clinical personnel cannot accurately quantify the effects of disordered breathing or drug administration on respiratory sufficiency. There is a need for a cost-effective, evidence-based, real-time solution to these challenges.

A novel, non-invasive respiratory volume monitor (RVM) has been developed that produces continuous digital volume traces and accurately reports minute ventilation (MV), tidal volume (TV) and RR in non-intubated patients. Continuous MV measurements provide direct assessment of respiratory function and can quantify apnea and respiratory deterioration in real time. Use of RVM technology is consistent with current American Society of Anesthesiologists guidelines for monitoring of ventilation in the PACU [10] and can provide health care professionals with a more complete assessment of respiratory status.

Here we report a case of a patient who experienced a substantial reduction in ventilation in response to a single opioid dose in the PACU. RR and pulse oximetry were insufficient to detect the potential threat to patient safety.

## Case presentation

**Figure 1:**
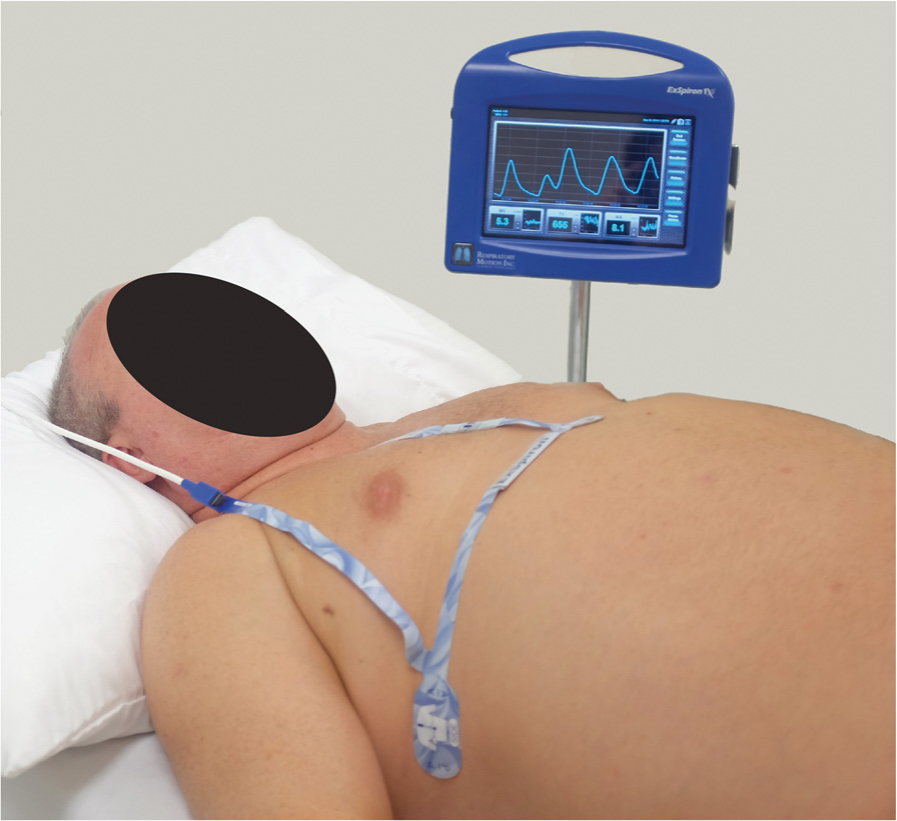
A non-invasive respiratory volume monitor (ExSpiron; Respiratory Motion, Inc.) that provides continuous, real-time, non-invasive measurements of minute ventilation, tidal volume and respiratory rate. This photograph shows standard electrode placement on an obese patient (not the patient reported here; body mass index, 36.7kg/m^2^). One electrode is placed at the sternal notch, another on the xiphoid and the third in the right mid-axillary line at the level of the xiphoid.

A 54-year-old Caucasian man (weight, 116kg; height, 185cm body mass index, 33.7kg/m^2^) underwent left total hip replacement surgery under general anesthesia. A bioimpedance-based RVM (ExSpiron, Respiratory Motion, Inc., Waltham, MA, USA) was used to collect digital respiratory traces via an electrode PadSet placed on the thorax (Figure 1), beginning pre-operatively and continuing until PACU discharge, for a total of 418 minutes (106 minutes pre-operatively, 192 intra-operatively and 120 minutes in the PACU). MV, TV and RR measurements were calculated every 5 seconds for the duration of this period from 30-second segments collected in a sliding window. The clinical staff were blinded to RVM data. Vital sign measurements and pulse oximetry readings were obtained as part of routine patient care and were available to caregivers for ongoing patient management.

**Figure 2:**
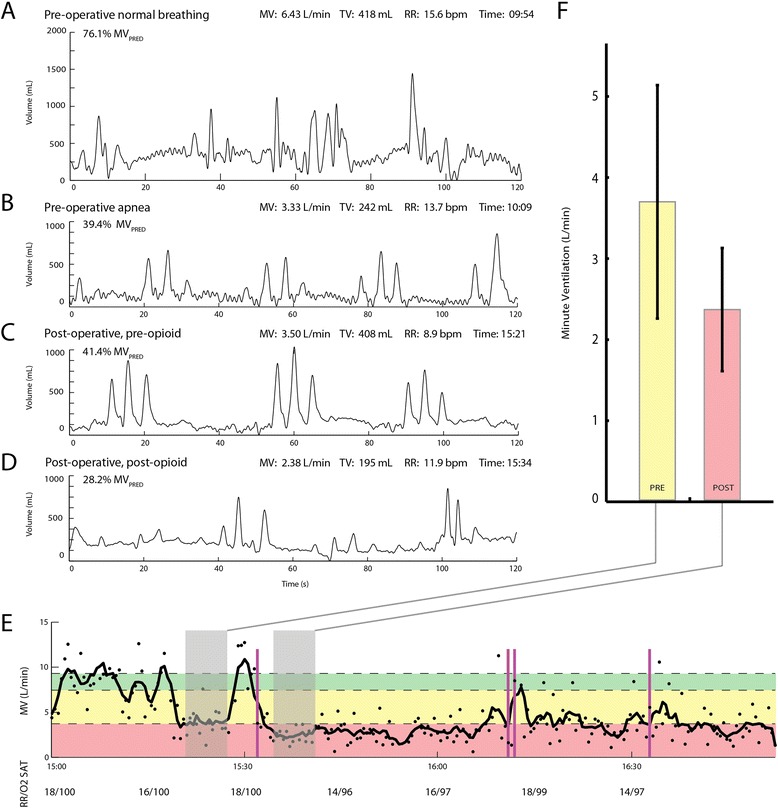
Two-minute captures of traces from a bioimpedance-based respiratory volume monitor over the course of the peri-operative stay, with average minute ventilation, tidal volume and respiratory rate. **(A)** Normal pre-operative breathing. **(B)** Pre-operative apnea. **(C)** Apnea prior to opioid administration in the post-anesthesia care unit (PACU). **(D)** Reduced ventilation with obstructed breathing after an initial opioid administration. Predicted minute ventilation (MV_PRED_) based on ideal body weight for the patient was 7.9L/min. **(E)** Time course of the patient’s minute ventilation (MV) over his entire PACU stay. Dashed horizontal lines represent (from top to bottom) 100%, 80% and 40% of MV_PRED_. Purple lines indicate opioid administrations (hydromorphone). Ventilation decreased following opioid administration and was persistently low until discharge. Respiratory rate (RR) and oxygen saturation levels, as documented by clinical personnel in the PACU flowchart, are shown below. **(F)** Mean and standard deviation for MV measurements recorded by the respiratory volume monitor (RVM) during 5 minutes of patient rest before and after an initial opioid administration (15:32) in the PACU (periods shown in gray in **(E)**). The RVM data depict a sudden drop in ventilation that is not reflected in either RR or oxygen saturation levels. TV, Tidal volume.

The patient’s past medical history was positive for OSA, with no other respiratory conditions. The patient owned a home model of a continuous positive airway pressure (CPAP) device but had not been using it. In the pre-operative holding area, the patient was very sleepy, though not yet sedated. The RVM trace showed sustained, visible manifestations of apnea over this period (Figure 2B). Clinical personnel had no way to observe these apneic episodes and were unaware of the patient’s disordered breathing. No snoring or physical indications of obstruction were observed. The patient was taken to the operating room, where sedation was induced with 300mg of propofol and 250μg of intravenous (IV) fentanyl and intubated after receiving 50mg of IV rocuronium. Two 0.5mg doses of hydromorphone were administered in the 15 minutes after incision, along with three additional doses of rocuronium (20mg, 10mg, 10mg) over the subsequent hour. Surgery lasted 134 minutes, after which the patient was extubated and transferred to the PACU.

Within 20 minutes of PACU arrival, prior to receiving any post-operative opioids, the patient began to exhibit apneic events lasting up to 45 seconds (Figure 2C). As in pre-operative holding, no outward indications of disordered breathing were observed. Using a standard formula based on ideal body weight [11], the patient’s predicted minute ventilation (MV_PRED_), expected to be sufficient to maintain blood oxygen and carbon dioxide levels under baseline conditions, was calculated to be 7.9L/min. Previous research has described ventilation below 80% of MV_PRED_ prior to opioid administration as putting a patient at risk for OIRD (MV <40% of MV_PRED_), a potential threat to patient safety [7]. Our patient’s average MV, as measured by the RVM during the 5 minutes of stable breathing prior to an initial PACU opioid administration, was 3.70L/min, 44% of MV_PRED_.

At this point, the patient received an isolated, nurse-administered dose of hydromorphone (0.4mg). This was followed by an immediate reduction in ventilation, with average MV of only 2.37L/min (28% of MV_PRED_) in the subsequent 5 minutes (Figure 2F). The patient’s RR gave no indication of compromise, remaining between 12 and 18 breaths per minute for the duration of this period and even showing a slight increase after hydromorphone administration (Figure 2E). Blinded to the RVM data and with no way to objectively evaluate ventilation, clinical personnel continued to administer opioids to manage pain in accordance with standard practice. The patient received a second nurse-administered hydromorphone dose (0.4mg) 40 minutes after the first. He was then provided with a patient-controlled analgesia (PCA) device and received three PCA doses (0.2mg hydromorphone each) in under 1 hour, before being discharged to a general care unit 118 minutes after PACU arrival (Figure 2E). MV remained low (<40% MV_PRED_) for the duration of the patient’s PACU stay and was still reduced upon discharge. RVM monitoring was discontinued at that time.

## Discussion

The events of this case illustrate the limitations of RR monitoring and pulse oximetry in the evaluation of post-surgical respiratory status. With a stable RR and no evidence of desaturation, our patient received opioids in the PACU in spite of already compromised ventilation. The compromised ventilation was not able to be immediately appreciated by clinical personnel. RR and oxygen saturation provided no forewarning of potential respiratory insufficiency, and the patient was treated with additional opioids, markedly increasing the risk for further respiratory decline. The patient was discharged to a general hospital floor without indication of the need for continuous respiratory monitoring, despite sustained low ventilation and additional opioid doses in the closing minutes of his PACU stay. Fortunately, he experienced no adverse events.

All clinical decisions in this case were made according to standard protocols. Any potential threat to patient safety was not the result of substandard care, but rather was due to inherent pitfalls in established respiratory monitoring technology and practice. In this case, the commonly used respiratory monitoring (RR and peripheral capillary oxygen saturation) did not suggest respiratory compromise, despite persistent low MV after opioid administration. If RVM data had been available, the patient’s initial respiratory depression would have been noted and the patient’s opioid regimen might have been adjusted or a multimodal approach to analgesia might have been pursued. Low MV readings and an RVM trace demonstrating disordered breathing characteristics might have led to the utilization of CPAP. Also, decreased MV at the time of discharge might have led clinical personnel to delay transfer to the floor or triage the patient to a step-down unit or monitored bed.

RR and pulse oximetry are only surrogate indicators of ventilatory drive. RR, as this case illustrates, does not reliably provide an accurate picture of overall ventilation, as it does not adequately reflect variations in tidal volume. Pulse oximetry has well-documented issues with reliability and false alarms in the clinical setting and represents at best only the end result of respiration [12-14]. As a result, there can be a substantial delay between onset of respiratory compromise and a detectable decline in oxygen saturation levels [12,13].

By comparison, the RVM’s reliable, continuous tracking of MV allows direct assessment of ventilatory drive in real time. Health care providers can quantify ventilation status on arrival to the PACU, recognize apnea, evaluate reductions in ventilation after opioid dosing, and prevent persistent respiratory compromise over the course of a PACU stay. The increased awareness afforded by the RVM facilitates the initiation of more timely interventions. Clinical personnel can modify opioid regimens, pursue alternative pain management strategies, or initiate CPAP or bilevel positive airway pressure. The RVM data also provide a valuable context for discharge and triage decisions, helping clinical personnel to avoid premature transfer and to select an appropriate acuity level for subsequent care. Individualization of the pain management regimen in the PACU and the ability to relay quantitative measurements upon transfer to the floor has the potential to enhance patient safety.

RVM technology may also have applications in pre-operative screening. Caregivers may have elected to manage the patient described in this report differently had they been able to identify and quantify the apnea exhibited prior to surgery. As discussed earlier, OSA presents a number of challenges to peri-operative care. The prevalence of OSA and its association with post-operative complications has led the American Society of Anesthesiologists and similar organizations to release guidelines calling for prolonged post-surgical observation of patients with OSA [15]. Hospitals are left with a management dilemma. Ignoring OSA may pose a threat to patient safety and lead to the involvement of costly higher acuity services, but generalized management programs can be cost-prohibitive as well and are potentially inefficient solutions. Non-invasive, real-time, continuous respiratory volume monitoring allows for direct, comprehensive assessment of individual ventilation during peri-operative care. This technology can contribute to continuing improvement of patient-specific protocols for the management of OSA, OIRD and other phenomena that pose a threat to respiratory competence in the aftermath of surgery.

## Conclusions

The events of this case illustrate the limitations of current respiratory rate monitoring and pulse oximetry in the evaluation of post-surgical respiratory status. Our patient displayed stable respiratory rate and no evidence of desaturation, despite sustained low minute ventilation, and he received opioids in the post-anesthesia care unit despite already compromised ventilation. Because the available monitoring did not indicate the patient’s true respiratory status, he was treated with additional opioids, markedly increasing his risk for further respiratory decline. Using the additional information provided by the RVM, care providers would be able to optimize post-operative care and improve patient safety.

## Consent

Written informed consent was obtained from the patient for publication of this case report and any accompanying images. A copy of the written consent is available for review by the Editor-in-Chief of this journal. Of note, the image in this report is not of the patient described here. Written informed consent was obtained for use of that patient’s image.

## Abbreviations

CPAP: Continuous positive airway pressure
IV: Intravenous
MV: Minute ventilation
MV_PRED_: Predicted minute ventilation
OIRD: Opioid-induced respiratory depression
OIVI: Opioid-induced ventilatory insufficiency
OSA: Obstructive sleep apnea
PACU: Post-anesthesia care unit
PCA: Patient-controlled analgesia
RR: Respiratory rate
RVM: Respiratory volume monitor
TV: Tidal volume
